# A Tunable Freeform-Segmented Reflector in a Microfluidic System for Conventional and Surface-Enhanced Raman Spectroscopy

**DOI:** 10.3390/s20051250

**Published:** 2020-02-25

**Authors:** Qing Liu, Michael Stenbæk Schmidt, Hugo Thienpont, Heidi Ottevaere

**Affiliations:** 1 Department of Applied Physics and Photonics, Brussels Photonics, Vrije Universiteit Brussel and Flanders Make, Pleinlaan 2, B-1050 Brussels, Belgium; liu.qing@vub.be (Q.L.); hthienpo@vub.ac.be (H.T.); 2 Department of Micro- and Nanotechnology, Technical University of Denmark, Ørsteds Plads, Building 345 east, 2800 Kgs. Lyngby, Denmark; Michael.Schmidt@nanotech.dtu.dk

**Keywords:** microfluidics, freeform surface, Raman spectroscopy, SERS, photonics, optical detection

## Abstract

We present a freeform-segmented reflector-based microfluidic system for conventional Raman and Surface-Enhanced Raman Scattering (SERS) analysis. The segmented reflector is directly designed by a numerical approach. The polymer-based Raman system strongly suppresses the undesirable background because it enables confocal detection of Raman scattering through the combination of a freeform reflector and a microfluidic chip. We perform systematic simulations using non-sequential ray tracing with the Henyey-Greenstein model to assess the Raman scattering behavior of the substance under test. We fabricate the freeform reflector and the microfluidic chip by means of ultra-precision diamond turning and laser cutting respectively. We demonstrate the confocal behavior by measuring the Raman spectrum of ethanol. Besides, we calibrate the setup by performing Raman measurements on urea and potassium nitrate solutions with different concentrations. The detection limit of our microfluidic system is approximately 20 mM according to the experiment. Finally, we implement a SERS microfluidic chip and discriminate 100 µM urea and potassium nitrate solutions.

## 1. Introduction

Raman spectroscopy is a powerful optical detection technique that was first discovered in 1928 by Sir C.V. Raman [1]. Nowadays it is widely used in biological research, pharmaceutics, chemical sciences and many other fields [2,3,4,5,6]. When a bundle of photons interacts with a molecule, the majority of the photons will be elastically scattered by the molecule as Rayleigh scattering, while a small portion (typically millionths) of the photons will be absorbed and re-emitted almost simultaneously by the molecule with a frequency shift due to the molecular vibrational modes. The inelastic scattering of the photons with energy shift is referred as Raman scattering, which can be captured and detected by a spectrometer as a Raman spectrum. The Raman spectra contain fingerprint information of the molecules under test that can be used for material characterization. Raman spectroscopy is suitable for label-free analysis of solids, liquids and gases qualitatively or quantitatively. Especially in recent years, the combination of a microfluidic chip with Raman spectroscopy has largely reduced the size and cost compared to traditional bulky Raman equipment [7,8,9]. One way to miniaturize the Raman system is to remove the lenses for collimation and focusing, but introduce optical fibers directly into the microfluidic chip for excitation and collection components [10,11,12,13]. The optical fiber-based Raman-on-chip systems are very compact. In such systems, the distance between the fiber facets and the fluid should be as small as possible, otherwise the excitation and collection efficiencies of the microfluidic chip will decrease significantly. One way to satisfy this requirement is to keep the fiber ends in the fluidic channel and be in contact with the sample directly. However, the analytes can contaminate the fiber facets easily during a measurement, therefore decreasing the reliability and stability of the Raman analysis. To prevent this, a compromise is to miniaturize some of the optical components into micro-optics and integrate them with microfluidic chip [14,15]. These optofluidic chips typically have higher sensitivity in comparison with the fiber-based microfluidic chips due to the higher excitation and collection efficiencies. We have reported a freeform reflector-based lab-on-chip for Raman spectroscopy with a noise-equivalent-concentration (NEC) for urea solution of 20 mM [14]. The lab-on-chip has suppressed the background of PMMA material—out of which the chip is fabricated—by a factor of 7. Nevertheless, the cost of an individual chip is very expensive since the freeform reflector embedded on the chip is fabricated via ultra-precision diamond tooling with considerably high manual and equipment requirements. In addition, the detection limit of our previous lab-on-chip with conventional Raman spectroscopy is not low enough to satisfy the requirements for biological and toxicological applications where the concentrations of the analytes are typically at ppm or even ppb level. Spectroscopy based on Surface-Enhanced Raman Spectroscopy (SERS) has been applied increasingly to reach higher detection sensitivity by integrating nanoparticles, nanodimers or specific SERS-biotags in the microfluidic systems for drug and cell detection [16,17,18,19]. With SERS, the Raman scattering of adsorbates can be enhanced by localized surface plasmon resonances (LSPR) on rough metal surfaces or nanostructures with noble metallic coating such as Au or Ag. The SERS enhancement factors are typically 10^4^–10^6^ and can be as high as 10^12^ for single molecule detection [20,21,22]. In this paper, we investigate a freeform reflector-based tunable Raman spectroscopy setup for microfluidic lab-on-chip analysis. The versatile Raman setup we built for the experimental proof-of-concept demonstration is compatible with our own PMMA-based lab-on-chip for conventional Raman and surface-enhanced Raman detection, as well as lab-on-chips that are commercially available.

## 2. Materials and Methods

### 2.1. Design of the Microfluidic Chip and Freeform Segmented Reflector

**Figure 1:**
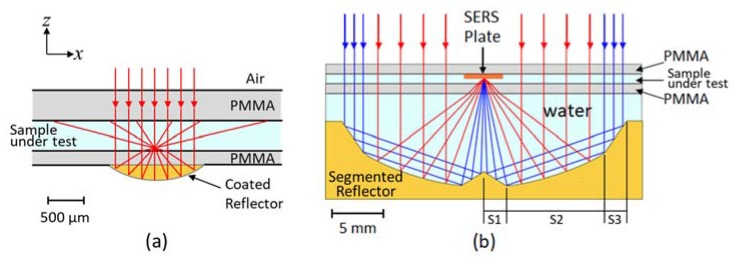
Side-view illustration of (**a**) our previously reported freeform reflector embedded lab-on-chip (NA = 1.24) [14] and (**b**) the segmented reflector-based Raman system with SERS microfluidic chip (NA = 1.15). The red and blue rays refer to the incident light that interact with different segments of the reflector. (S1: center segment; S2: middle concave segment; S3: marginal segment).

Our previous reported lab-on-chip consists of three layers [14], as shown in Figure 1a. The 200 µm thick bottom layer contains a freeform reflector with 200 nm gold coating to focus the excitation beam and collect the scattered Raman signal. The middle layer is a 500 µm thick fluidic layer for the sample flow. The top PMMA layer is the sealing layer for the fluidic channel including in- and outlets.

The focal point of the freeform reflector is located in the fluidic layer 50 µm above the interface between the fluidic and the reflector layer. The fixed freeform reflector on chip enables robust confocal Raman detection but has no flexibility to introduce SERS, since the SERS effective area is typically close to the surface of the metallic surface in a range smaller than 100 nm. Moreover, the SERS substrates are covered with a metallic coating and therefore opaque. The incident beam and the Raman scattered light will be blocked in the reflector-based system when introducing the SERS substrate without any modification. 

To this end, we bring forward a segmented mirror design that work together with a microfluidic chip, by which the majority of the excitation light can be utilized and all the scattered light within a certain field can bypass the SERS substrate and can be collected. The microfluidic chip consists of three layers, including the PMMA-based bottom and top sealing layers and a channel layer for the fluidic samples under test, as shown in Figure 1b. Each PMMA layer has a thickness of 1 mm. The reflector is composed of three segments, the center segment, the middle concave segment and the marginal segment at the outer rim of the reflector. In the excitation path, the majority of excitation light will be directly reflected to the microfluidic channel by the middle segment. The outer part of the beam will be firstly reflected by the marginal segment and then focused by the center segment to the same focal point as that of the concave segment. Therefore, only a small amount of the excitation energy is blocked by the SERS substrate. And vice versa, in the collection path, the center segment and the marginal segment provide the Raman signal collection with two reflective surfaces and can work together to collimate the Raman scattering close to the optical axis. At the same time the middle segment can collimate the rest of the Raman scattered light towards the same direction. The profile of our freeform segmented reflector is calculated via a numerical approach based on Fermat’s principle [23]. Our segmented reflector has a numerical aperture of 1.15 and a diameter of 30 mm. The tunability of our segmented reflector is 4 mm. The details of the numerical approach are available in the Supporting Information (Figures S1–S3).

### 2.2. Non-Sequential Simulation

We performed a non-sequential ray tracing simulation for our system using the Henyey-Greenstein model in OpticStudio (Zemax, Kirkland, WA, USA). This model was initialized by Henyey and Greenstein in 1941 to describe the angular distribution of light scattered by interstellar matter [24]. Nowadays it has been applied as one of the typical Rayleigh models in various situations, ranging from the scattering of light by chemical emulsions [25], biological tissues [26] to atmospheric and interstellar clouds [27]. Since Raman scattering has common features with Rayleigh scattering, the intensity of scattered light is inversely proportional to the fourth power of the incident light and the size of particles inducing the scattering is much smaller than the wavelength of the excitation, we use this model for our non-sequential simulations to mimic the interaction of the excitation light with the PMMA chip and sample undertest. Details of the non-sequential ray tracing based on the angular distribution of Henyey-Greenstein model [24,28] and OpticStudio settings [29] can be found in the Supporting Information.

**Figure 2:**
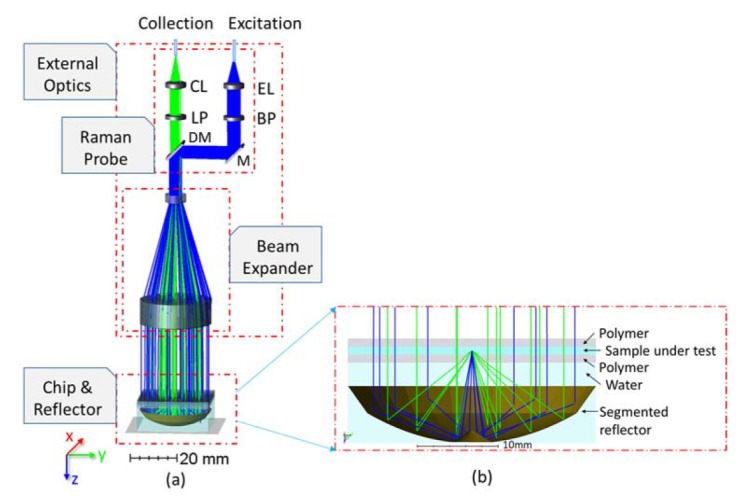
Non-sequential simulation in OpticStudio. (**a**) Overall layout, and (**b**) the microfluidic chip and segmented reflector. The blue rays and green rays refer to the excitation light and Raman light respectively. (EL: Excitation lens; CL: Collection lens; BP: Band-pass filter; M: Mirror; LP: Long-pass filter; DM: Dichroic mirror).

In addition to the microfluidic chip and segmented reflector, the microfluidic system also contains external optics consisting out of an excitation path and a collection path (Figure 2). In the excitation path, the excitation laser coming out of the facet of the excitation fiber is collimated into a parallel beam by the excitation lens. A band-pass filter (BP) is used to suppress the laser intensity at other frequencies. The mirror (M) and dichroic mirror (DM) then reflect the collimated beam to a beam expander consisting out of a concave and a convex lens. The expanded laser beam with the same diameter of the segmented reflector passes through the microfluidic chip and focuses inside the fluidic channel by the segmented reflector. 

In the collection path, the generated Raman scattering is collimated by the reflector and bypass the central region of the microfluidic chip. Then the Raman beam is reduced in diameter by the beam expander, passes through the dichroic mirror and the long-pass filter, is collected into the collection multi-mode fiber by the collection lens.

In the non-sequential simulations, the wavelength of excitation is 785 nm, and the wavelength of scattering is 850 nm, which is close to the fingerprint Raman bands of ethanol, urea and potassium nitrate. These chemicals are also the analytes we will use in the following experiments. For the microfluidic chip, the channel layer with fluidics, as well as the two polymer layers can be defined as scattering volumes respectively in accordance with the Henyey-Greenstein model. The thickness of each layer is 1 mm.

We perform the non-sequential ray tracing simulations to evaluate three characteristics of our Raman system: confocality, background suppression, and alignment tolerance of external optics with respect to the reflector.

Our Raman system is a confocal system, in which the collection fiber works as the pinhole to attenuate the out-of-focus scattering. In this confocal system, only the scattering close to the focus will be collected into the multimode fiber (MMF). Scattering from above or below the focal point is regarded as background and will be suppressed due to the proper choice of the diameter of the multi-mode fiber. 

**Figure 3:**
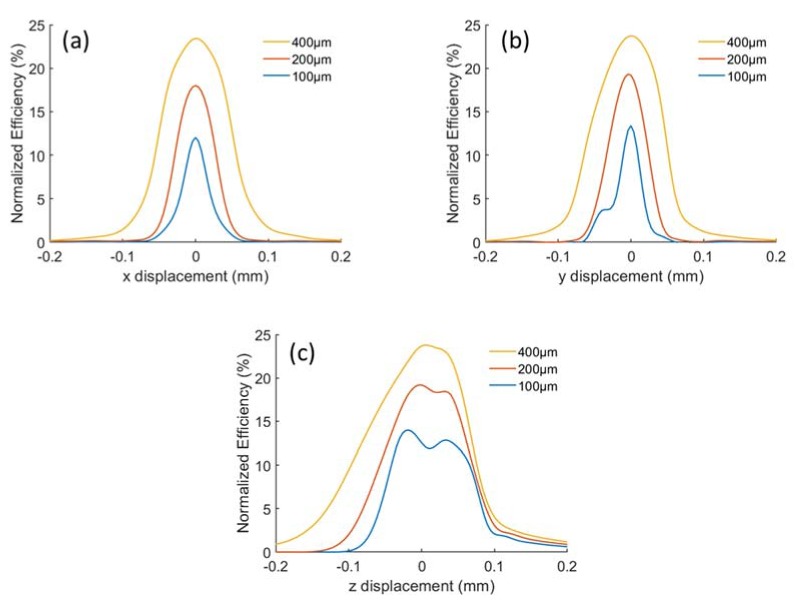
Confocal behavior of the system by simulation. Each subfigure shows the efficiency changes with respect to the displacement of the point source in (**a**) x-, (**b**) y- and (**c**) z-axis, respectively. 100 µm, 200 µm and 400 µm MMF are used as collection fiber.

To evaluate the confocality, we place a point source in the fluidic channel to demonstrate the Raman source from a molecule. The light from this point source can be received by the MMF through the collection path. By displacing the point source out of the optimal position in different directions and detect the flux received on the fiber tip, we can get the confocality curve, as shown in Figure 3. MMFs with diameters of 100, 200 and 400 µm are modeled in our simulation. The collection efficiency is defined as the proportion of Raman scattered light that can be collected by our system over the entire 4π steradian of all Raman scattered light. If all backward Raman scattering can be collected, the normalized efficiency should be 50%. According to the simulation, a maximum normalized efficiency of 23.8% can be realized by our Raman setup when using a 400 µm MMF. After fitting the confocality curve with a normal distribution, we can obtain the Full Width Half Maximum (FWHM) of the flux change with respect to the displacement of the point source, as listed in Table 1.

From the simulation results, we notice that a larger core diameter of multi-mode fiber leads to a higher collecting efficiency, but a lower confocality with larger FWHM. It is reasonable that more scattering from both the in-focus and out-of-focus regions are collected with a larger core size. Besides, two extrema appear on the confocality curve at the z direction. This is because the parameters of all the optical components for simulation as well as experiments are from commercial companies, and the aberrations when they are aligned together are relatively large, resulting in non-parallel rays towards the reflector, thereby giving rise to separated focal points for different segments of the reflector. This phenomenon is more obvious when the diameter of MMF is small. Therefore, we should select a suitable fiber to get a trade-off between efficiency and confocality for different scenarios.

Confocality refers to the system’s ability to suppress the background outside the region of interest. However, it is very difficult to measure this parameter experimentally because no perfect point source is available in reality. Therefore, we use another related parameter, the suppression factor (*SF*), to represent the confocality that is both theoretically and experimentally obtainable.

For the simulation of the confocal performance, we define the top, bottom and the channel layer as scattering volume by the Henyey-Greenstein model. The Raman intensity observed at the collection fiber from these layers are $I_{t},\;I_{b}$ and $I_{m}$ respectively with identical excitation intensity. The suppression factor (*SF*) accordingly can be calculated via the following equation in the simulation:

(1) $$SF=I_{m}/\left(I_{t}+I_{b}\right)$$

As our setup includes the segmented reflector, the microfluidic chip as well as the external optics, the alignment of the different parts can be critical. Especially when the collection fiber in the Raman probe plays the role as the pinhole in the confocal system, a small displacement of the MMF can reduce the Raman intensity obtained. To assess the influence of misalignments, we displace the MMF in x, y and z directions and detect the flux collected by the fiber. Simulation results are compared with the experimental results in Section 3.2.

The non-sequential ray tracing simulations also demonstrate that the segmented reflector can collect 20% more Raman scattering compared to a simple concave mirror in combination with a 4 mm by 4 mm SERS substrate inside the fluidic chamber. In addition, ideally, the spot diameter of the excitation light on the focal plane could reach the diffraction limit as our segmented reflector is designed via a numerical approach such that all excitation rays can be focused in a common point. However, this goal is difficult to achieve because of the aberrations of the external optics used. According to our non-sequential ray tracing simulation with an optimized layout of all optical components, 75% of the tracing rays fall within a 5 µm diameter circle on the focal plane. The spot size of the excitation light could be further minimized by using customized optical components with less aberrations. 

### 2.3. Fabrication of the Segmented Reflector and the Microfluidic Chip

**Figure 4:**
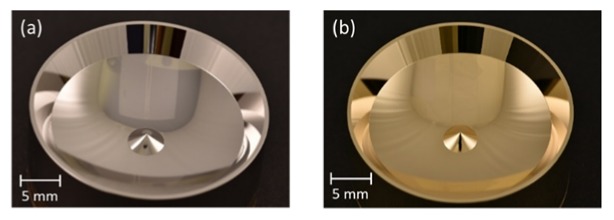
NiP surface of segmented reflector by ultra-precision diamond turning (**a**) and after gold coating (**b**). The reflectivity increases from 53% to 95% for NIR light when adding the Au coating.

After the design and simulation of the segmented freeform reflector, we fabricated the segmented reflector and the microfluidic chip separately with different approaches. The base material of the segmented reflector is brass. It was pre-fabricated to the approximate shape of the segmented reflector and coated with a nickel phosphorus (NiP) layer by electroless nickel plating. Then we use ultra-precision diamond turning to create the segmented surface. NiP layer can increase the hardness, corrosion and wear resistance for the segmented reflector when immersing in water [30]. But the reflectivity of NiP is only 53% for NIR light. Therefore, we finalize the segmented reflector by placing a 50 nm thick Au layer using sputtering coating. The reflectivity after coating is larger than 95% for the NIR light. The brass/NiP approach with Au coating makes it easy to clean the surface with isopropyl alcohol (IPA) or acetone after an experiment. Figure 4 shows the NiP surface of our segmented reflector and the surface after gold coating. 

The surface roughness and profile of our segmented reflector are measured with a non-contact profilometer (Contour GT-I, Bruker, Billerica, MA, USA) and a multisensor coordinate measurement machine (VideoCheck UA400 with Werth Fiber Probe, Werth Messtechnik, Giessen, Germany), respectively. The RMS roughness of the gold coated surface is 14.7 ± 1.4 nm when measuring 10 different areas of 200 µm × 200 µm. The standard error of the surface profile is less than 2 µm, close to the detection limit of the Werth Fiber Probe used.

For the microfluidic chip, the three layers are fabricated from PMMA polymer by laser cutting. The width of the channel is 600 µm, and the detection chamber in the center of the middle layer has a diameter of 6 mm. The minimum volume consumption of our microfluidic chip for the experiment is 30 µL. Then we use UV curing adhesive to bond these three layers together.

**Figure 5:**
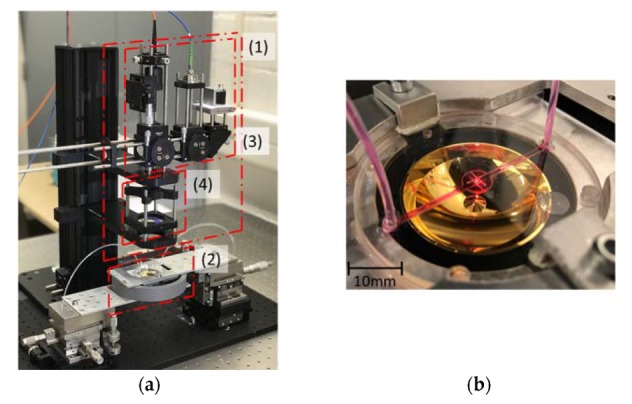
Proof-of-concept demonstration setup of our Raman spectroscopy system (**a**), (1): the external optics, (2): Microfluidic chip and segmented reflector, (3): Raman probe, (4) beam expander; Microfluidic chip and segmented reflector in detail (**b**).

We implement a proof-of-concept setup consisting out of external optics, a segmented reflector and microfluidic chip, as shown in Figure 5. We use a diode laser (TEC-500-0785-300, Sacher Lasertechnik, Marburg, Germany) with a maximum laser power of 300 mW to emit 785 nm wavelength light for excitation, and a spectrometer (AvaSpec-HERO, Apeldoorn, The Netherlands) to obtain the Raman signals for collection. The spectrometer is equipped with a 1024 × 58 pixel back illuminated TE cooled CCD detector and a blaze grating with grooves of 830 L/mm for the 788–1020 nm wavelength range. The slit width of the spectrometer is 25 µm corresponding to a spectral resolution of 0.7 nm. The conventional Raman spectra showed are obtained with an excitation power of 150 mW and an acquisition time of 15 s. The SERS measurement is performed with an excitation power of 70 mW and an acquisition time of 5 s.

### 2.4. Preparation of the SERS Substrates and SERS Chip

**Figure 6:**
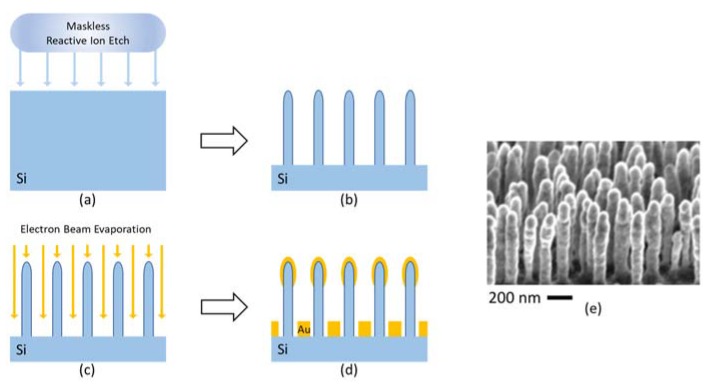
The fabrication process of SERS substrate. (**a**,**b**) Maskless reactive ion etching forms silicon nanopillars; (**c**,**d**) Electron beam evaporation forms Au layers; (**e**) SEM image of the nanopillars with coatings [31].

The SERS substrate is fabricated with the combination of maskless reactive ion etching and electron beam evaporation [31], as shown in Figure 6. 

An undoped single crystal silicon wafer with a diameter of 4 inches is processed with an Advanced Silicon Etcher (Surface Technology Systems MESC Multiplex ICP, Newport, UK) to form a large area of aperiodic nano-pillars with a pillar width of 50–80 nm and a height around 600 nm. A 200 nm thick Au layer is placed on top of the nano-pillars with electron beam evaporation (SCM 600, Alcatel, Annecy, France). The processed wafer is then laser cut into 4 mm by 4 mm pieces for SERS analysis. The enhancement factor of this SERS substrate can be as large as 2.4 × 10^6^ [31].

**Figure 7:**
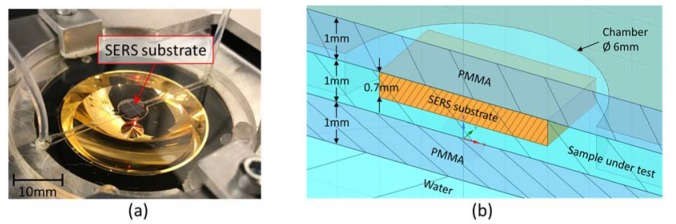
(**a**) Microfluidic chip integrated with SERS substrate to enhance the sensitivity of the Raman analysis. (**b**) Cross-section of the SERS microfluidic Chip.

We bond the backside of the SERS substrate on the top PMMA layer by UV curing adhesive. The SERS substrate is then integrated inside the microfluidic chamber by bonding the PMMA-based channel layer and bottom layer together with UV curing adhesive, as shown in Figure 7. By tuning the height of the segmented reflector, we place the focal point right on the surface of SERS substrate to obtain the best enhancement.

## 3. Results and Discussion

### 3.1. Suppression Factor

**Figure 8:**
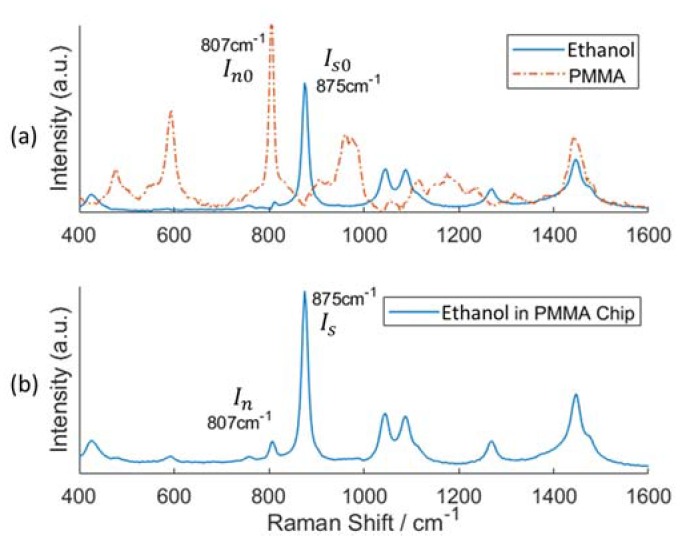
(**a**) Raman spectra of ethanol and PMMA measured by commercial Raman microscope (Bruker Senterra at the Department of Chemistry of Ghent University), and (**b**) Raman spectrum of ethanol in the PMMA chip obtained with our setup using a 200 µm diameter MMF fiber. The PMMA background is suppressed with a factor of 6. (figures are in the same scale).

Because our microfluidic chip is made from PMMA material, we will not only collect Raman scattering but also the background signal from PMMA. We use ethanol to determine the experimental suppression factor. The initial Raman spectra of ethanol and PMMA can be measured by commercial Raman spectroscopy (Figure 8a). Ethanol has a very strong Raman response at around 875 cm^−1^. We assume its peak value is $I_{s0}$ at this Raman shift. The Raman signal of PMMA is significantly strong at 807 cm^−1^, and its peak value is $I_{n0}$ under the same criterion as for the ethanol detection. The Raman spectrum obtained by our setup has peak values of $I_{s}$ and $I_{n}$ at 875 cm^−1^ and 807 cm^−1^, respectively. The experimental suppression factor can be calculated as:

(2) $$SF=\left(\frac{I_{s}}{I_{n}}\right)*\left(\frac{I_{s0}}{I_{n0}}\right)$$

**Figure 9:**
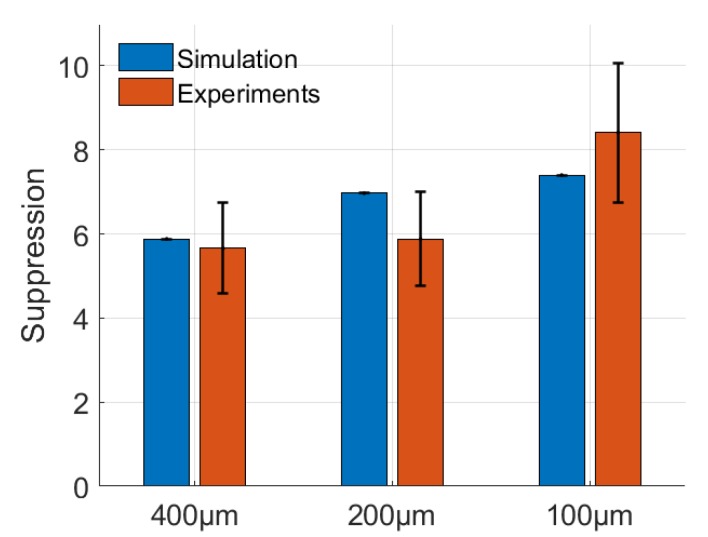
Comparison of the suppression factors obtained by simulations and experiments. The average and standard deviation of the experimental suppression factor for each MMF is calculated over 15 measurements.

The term $I_{s0}/I_{n0}$ is added in the equation to normalize the Raman intensity of different substances. As shown in Figure 8b, in our confocal setup, the background from PMMA is suppressed significantly compared to the Raman spectrum of PMMA. The comparison of the suppression factors obtained by experiments and simulations is shown in Figure 9. Same as in the simulation, we use different MMFs with core diameters of 100, 200 and 400 µm. The smaller the core diameter of the MMF, the larger the suppression factor, therefore we obtain better confocality. However, in this case the amount of light coupled into the collection fiber decreases. The suppression factor for the 400 µm and 200 µm MMF is around 6. And the suppression factor for the 100 µm MMF is over eight in our proof-of-concept setup. 

### 3.2. Alignment Tolerance Analysis

**Figure 10:**
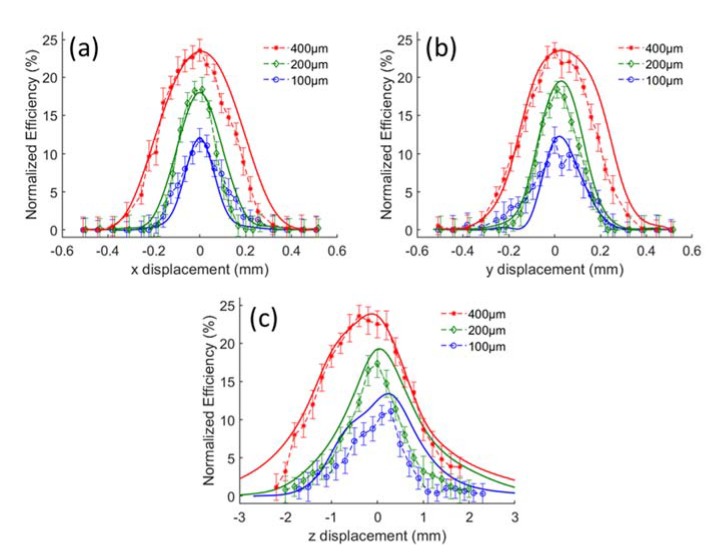
Raman efficiency of our setup with respect to misalignments of the MMF along (**a**) x-, (**b**) y-, (**c**) z-axis, respectively. 100, 200 and 400 µm MMFs are used.

For analyzing the alignment tolerances we also use ethanol as analyte. By adjusting the position of the collection fiber in the x-, y- and z-direction as shows in Figure 2 and obtaining the corresponding peak values of the Raman spectrum at 875 cm^−1^, we get the alignment tolerance curves as shown in Figure 10. The average and standard deviation of each point in the curve are obtained over 10 measurements. All of the peak values are normalized over 4π steradian to compare with the simulation results. The results from simulations and experiments are in good agreement, which approves the validity of our non-sequential ray-tracing approach using the Henyey-Greenstein model. We use the FWHM of the alignment tolerance curve to determine the misalignment allowance. Both the simulated and experimental misalignment allowance in the horizontal direction is approximately 150, 220 and 380 µm for the 100, 200 and 400 µm MMF, respectively. The misalignment allowance in the z-direction is 1.9, 2.1 and 2.7 mm for the 100, 200 and 400 µm MMF in the simulations. According to the experiments, while the allowance in the z-direction is 1.5, 1.5 and 2.3 mm, respectively. As expected, the misalignment allowance in the vertical direction is larger than that in the horizontal direction. We also note that the symmetry of the alignment tolerance curve in the horizontal direction is better than in the vertical direction. This is due to the aberrations of the external optics and the resulted splitting of the focal points of different segments, which we have discussed in the confocality analysis.

### 3.3. Limit of Detection for Conventional Raman Analysis

We used two kinds of analytes, urea and potassium nitrate (KNO_3_), to evaluate the sensitivity or the detection limit of the setup for conventional Raman. Urea is a common simple-structure organic compound with chemical formula CO(NH_2_)_2_. It is one of the metabolites of animal cells and is therefore used as an important indicator in many biological and medical tests. Urea has a strong Raman scattering near 1000 cm^−1^. Potassium nitrate is one kind of inorganic salt widely used in the industry. The Raman peak of KNO_3_ is located around 1040 cm^−1^.

In the sensitivity experiments, we set the integration time to 15 s, and power of excitation into the microfluidic chip to 150 mW. The wavelength of the excitation is 785 nm as before. We take 10 measurements for each solution to obtain the Raman spectra.

**Figure 11:**
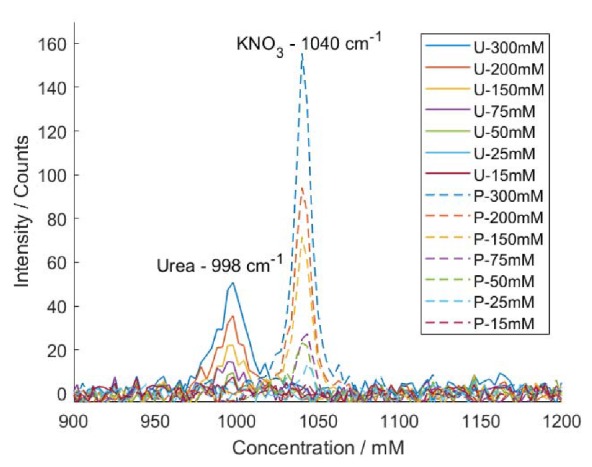
Raman spectra of aqueous urea (U) and KNO_3_ (P) solution with a concentration from 15 to 300 mM. The baseline is corrected by subtracting the reference spectrum of water. Each spectrum is obtained over 10 measurements.

**Figure 12:**
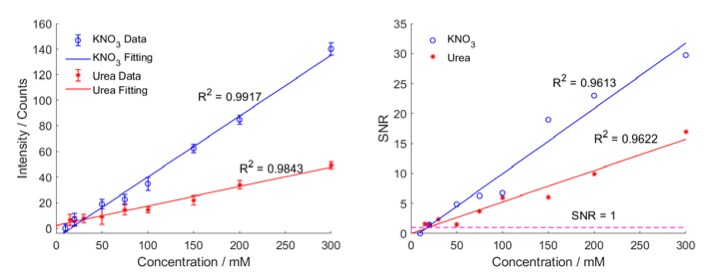
(**a**) Calibration curves and (**b**) SNR curves of aqueous urea and potassium nitrate solutions. The average and standard deviation for each solution are obtained over 10 measurements. According to the SNR curves, the NEC of our setup for urea and KNO_3_ are 19 and 18 mM, respectively.

First, deionized water was injected into the microfluidic channel and its Raman spectrum was taken as a reference for the following measurements, as shown in Figure S4 in the Supporting Information. Then, the Raman spectra of a series of aqueous urea and KNO_3_ solutions from 15 to 300 mM were measured, as shown in Figure 11. After subtracting the reference, we obtained the average spectrum and standard deviations for each solution. To reduce the impact of data fluctuations and spectral resolution, the mean peak value of urea and KNO_3_ is calculated by taking the mean value of the spectrum data of urea and KNO_3_ around 1000 cm^−1^ and 1040 cm^−1^, respectively. The spectra of urea and potassium nitrate as well as their main bands are shown in Figures S5 and S6 in the Supporting Information. When we plot the Raman intensity of both urea and KNO_3_ as function of concentration, we get the calibration curve (Figure 12a). The signal-to-noise ratio (SNR) for each concentration can be obtained by dividing the average Raman intensity of each solution by the standard deviation, as shown in Figure 12b. In a next step, the sensitivity of our setup for urea and KNO_3_ can be accessed by the noise-equivalent-concentration (NEC). NEC is the relative concentration in which its Raman intensity of interest equals to the noise, or SNR = 1. According to the SNR curve, the NEC of our setup for urea and KNO_3_ are approximately 19 and 18 mM, respectively. However, it should be noted that the limit of quantification of our microfluidic system could be at a much higher concentration than the NEC. However, it should be noted that in order to reliably detect the analyte and meet some predefined goals for bias and imprecision, the limit of quantification of our microfluidic system could be at a much higher concentration than the NEC.

### 3.4. Using the Microfluidic Chip and Segmented Reflector in Combination with a SERS Substrate

**Figure 13:**
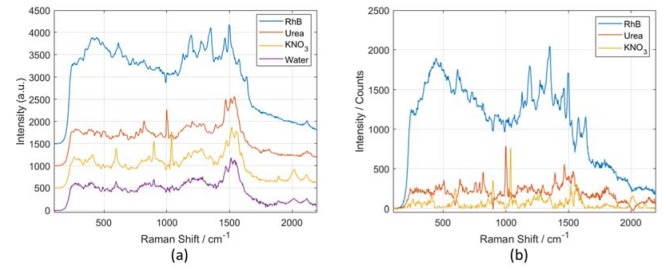
(**a**) SERS spectra of Rhodamine B, urea, potassium nitrate and water sample obtained with our microfluidic chip in combination with maskless ion etched SERS substrates, (**b**) the baseline corrected SERS spectra of Rhodamine B, urea and potassium nitrate by subtracting water as reference. Each spectrum is obtained over 10 measurements.

The aforementioned experimental results show that the limit of detection for aqueous solution with conventional Raman is roughly 20 mM, which cannot meet the requirements of many biological and chemical applications. Therefore, we perform SERS measurement with our microfluidic chip in combination with the etched SERS substrate. In the SERS measurements, we still use 785 nm wavelength laser as excitation source, but reduce the power of excitation into the chip from 150 to 70 mW and decrease the integration time from 15 s to 5 s to avoid damage of the Au coated nanopillars. The SERS spectra of 0.1 mM (6 ppm) urea solution and 0.1 mM (10 ppm) KNO_3_ solution are shown in Figure 13a. By subtracting the background SERS signal of water we obtain the baseline corrected SERS spectra of urea and KNO_3_, as shown in Figure 13b. The SERS spectra of different analytes are available in the Supporting Information (Figures S7 and S8). The SERS measurements show that even with a lower power and a shorter integration time, the peaks of 0.1 mM analytes can be observed clearly by the SERS chip. The 0.1 mM concentration of urea solution we tested with our SERS chip is 6.7 times better than the result in literature [32]. The 0.1 mM concentration of KNO_3_ detected with our SERS chip is a bit larger than the 1ppm limit of detection reported [33]. This can be partially explained by a longer integration time (60 s) used. Nevertheless, the ability of our SERS chip for quantitative analysis needs to be further investigated. We also measured the SERS spectrum of 10 µM Rhodamine B (RhB) solutions with our SERS chip. The Raman peaks of RhB at 612, 1130, 1274, 1351 and 1499 cm^−1^ can be clearly discriminated. From the SERS-based experimental results, we observe that RhB is more sensitive than urea and KNO_3_. The main reason is a higher cross-section of RhB. In addition, the SERS enhancement can also be largely impacted by the assimilation of molecules with the surface of the SERS substrate; RhB molecules can easily adsorb on the SERS substrate, thus inducing a stronger Raman response of RhB sample. The main SERS bands and their relative assignments of molecular vibrational modes are listed in Table 2. However, it must be noted that the SERS spectra of water and urea present several abnormal Raman peaks and bands in the regions around 600, 900, 1500 and 2000 cm^−1^. The presence of these abnormal Raman bands is likely to be related to the impurity of the analytes and the contamination during the sample preparation as well as the experiments. As a result, other molecules rather than the analyte with high assimilation and Raman cross-sections could generate amplified Raman signals by the high enhancement factor (~10^6^) of our SERS substrate and are as such added to the spectrum of the analyte.

## 4. Conclusions

We designed a freeform-segmented reflector-based microfluidic system for conventional Raman and SERS measurements. The surface profile of our freeform-segmented reflector is calculated by a numerical approach. We performed the non-sequential ray tracing simulations in OpticStudio by using the Henyey-Greenstein model to define the scattering volumes. The confocality, alignment tolerances of the external optics with respect to the microfluidic chip system and background suppression performance of our setup have been simulated. We fabricated the segmented reflector and microfluidic chip and implemented a proof-of-concept setup. The experimental results obtained with our setup are in good agreement with the simulation results, which proves the feasibility of our simulation approach.

The NEC of our setup for aqueous urea and KNO_3_ solution is approximately 20 mM, comparable to the value obtained with our previously reported lab-on-chip setup [14]. To further increase the sensitivity of our Raman system, we integrated a SERS substrate into the channel of microfluidic chip. The SERS microfluidic chip in combination with our Raman setup is capable to discriminate 0.1 mM urea and KNO_3_ solutions, which is compatible with the literature results [32,33]. Our SERS microfluidic chip is also able to detect the Raman peaks of RhB solution with a concentration of 10 µM. The SERS microfluidic chip has extended potential applications of our setup to the biological and chemical application domain that require a lower limit of detection.

However, it should be noticed that the Raman enhancement effect is highly correlated with the number of molecules adsorbed on the nanostructures. As the adsorption of different molecules varies widely, the Raman enhancement for various analytes are quite different even if the samples have the same concentration. The uniformity of the SERS substrate also greatly affects the Raman response. For the moment, the existence of compounds in an aqueous solution with low concentration can be determined with our setup. Nevertheless, the sensitivity of our SERS microfluidic chip can be further improved by optimizing the optical components in the external optics and improving the uniformity of the SERS substrate. Finally, the quantitative analysis capabilities of our system for SERS measurements should in future be investigated using p-MBA and biological samples.

## Figures and Tables

**Table 1:** Confocality behavior of the setup from simulations.

MMF (µm)	Max Efficiency	Confocality - FWHM (µm)		
		x	y	z
**100**	13.4%	41.8	35.2	120.0
200	19.3%	57.8	57.4	124.6
400	23.8%	100.0	100.4	155.6

**Table 2:** Main SERS bands of urea, potassium nitrate and Rhodamine B and their relative intensities and assignments (ν: stretching, δ: deformation; S: strong, M: medium, W: weak).

Analyte	Literature (cm^−1^)	SERS (cm^−1^)	Assignment
Urea [34]	1012 S	1000 S	ν (N-C-N)
	1474 W	1467 M	ν (N-C-N)
	1542 W	1540 M	δ (NH_2_)
Potassium nitrate [35]	1050 S	1040 S	ν (NO_3_)
	1343 W	1357 W	ν (NO_3_)
Rhodamine B [36,37]	619 S	615.9 S	ν (Aromatic C-C)
	1130 W	1130 W	δ (Aromatic C-H)
	1199 M	1190 S	δ (Aromatic C-H)
	1284 S	1274 M	δ (C-C)
	1360 S	1351 S	ν (Aromatic C-C)
	1508 S	1499 S	ν (Aromatic C-C)
	1591 W	1581 S	ν (C=C)
	1644 S	1638 S	ν (Aromatic C-C)

## Supplementary Materials

The following are available online at https://www.mdpi.com/1424-8220/20/5/1250/s1, Figure S1: Surface profile calculation of the middle concave segment of the freeform-segmented reflector, Figure S2: Schematic design of all segments of the reflector, Figure S3: Two configurations of the reflector and microfluidic chip design, Figure S4: Raman response of water and ethanol in the fluidic channel, Figure S5: Raman spectra of urea and potassium nitrate solutions with different concentrations, Figure S6: Main Raman bands of urea and potassium nitrate, Figure S7: Surface enhanced Raman spectra of 0.01 mM Rhodamine B, 0.01 mM urea, 0.01 mM potassium nitrate solutions and water, Figure S8: Baseline corrected Raman spectra of 0.01 mM Rhodamine B (RhB), 0.1 mM urea, 0.1 mM potassium nitrate solutions obtained with our microfluidic system in combination with the SERS substrate.

## References

[1] Raman C.V. (1928). A new radiation. India J. Phys..

[2] Tu Q., Chang C. (2012). Diagnostic applications of Raman spectroscopy. Nanomed. Nanotechnol. Biol. Med..

[3] Lim I.-I.S., Njoki P.N., Park H.-Y., Wang X., Wang L., Mott D., Zhong C.-J. (2008). Gold and magnetic oxide/gold core/shell nanoparticles as bio-functional nanoprobes. Nanotechnology.

[4] Aoki P.H.B., Furini L.N., Alessio P., Aliaga A.E., Constantino C.J.L. (2013). Surface-enhanced Raman scattering (SERS) applied to cancer diagnosis and detection of pesticides, explosives, and drugs. Rev. Anal. Chem..

[5] Matteini P., Cottat M., Tavanti F., Panfilova E., Scuderi M., Nicotra G., Menziani M.C., Khlebtsov N., de Angelis M., Pini R. (2017). Site-Selective Surface-Enhanced Raman Detection of Proteins. ACS Nano.

[6] Krafft C., Popp J. (2015). The many facets of Raman spectroscopy for biomedical analysis. Anal. Bioanal. Chem..

[7] Habermehl A., Strobel N., Eckstein R., Bolse N., Mertens A., Hernandez-Sosa G., Eschenbaum C., Lemmer U. (2017). Lab-on-chip, surface-enhanced Raman analysis by aerosol jet printing and roll-to-roll hot embossing. Sensors.

[8] Liu L., Devoe D.L., White I.M. Microfluidic SERS Using a 3-Dimensional Porous Monolith as a SERS-Active Solid Phase in a Microchannel. Proceedings of the Conference on Lasers and Electro-Optics 2010.

[9] Uusitalo S., Hiltunen J., Karioja P., Siitonen S., Kontturi V., Myllylä R., Kinnunen M., Meglinski I. (2015). Performance and flow dynamics studies of polymeric optofluidic sers sensors. J. Eur. Opt. Soc..

[10] Khijwania S.K., Tiwari V.S., Yueh F.Y., Singh J.P. (2007). A fiber optic Raman sensor for hydrocarbon detection. Sens. Actuators, B Chem..

[11] Ashok P.C., Singh G.P., Tan K.M., Dholakia K. (2010). Fiber probe based microfluidic Raman spectroscopy. Opt. Express.

[12] Ashok P.C., Singh G.P., Rendall H.A., Krauss T.F., Dholakia K. (2011). Waveguide confined Raman spectroscopy for microfluidic interrogation. Lab Chip.

[13] Dochow S., Beleites C., Henkel T., Mayer G., Albert J., Clement J., Krafft C., Popp J. (2013). Quartz microfluidic chip for tumour cell identification by Raman spectroscopy in combination with optical traps. Anal. Bioanal. Chem..

[14] De Coster D., Loterie D., Ottevaere H., Vervaeke M., Van Erps J., Missinne J., Thienpont H. (2015). Free-form optics enhanced confocal Raman spectroscopy for optofluidic lab-on-chips. IEEE J. Sel. Top. Quantum Electron..

[15] Wang W., Zhao J., Short M., Zeng H. (2015). Real-time in vivo cancer diagnosis using Raman spectroscopy. J. Biophotonics.

[16] Zhou Q., Meng G., Zheng P., Cushing S., Wu N., Huang Q. (2015). A Surface-Enhanced Raman Scattering Sensor Integrated with Battery-Controlled Fluidic Device for Capture and Detection of Trace Small Molecules. Sci. Rep..

[17] Perozziello G., Candeloro P., De Grazia A., Esposito F., Allione M., Coluccio M.L., Tallerico R., Valpapuram I., Tirinato L., Das G. (2016). Microfluidic device for continuous single cells analysis via Raman spectroscopy enhanced by integrated plasmonic nanodimers. Opt. Express.

[18] Kline N.D., Tripathi A., Mirsafavi R., Pardoe I., Moskovits M., Meinhart C., Guicheteau J.A., Christesen S.D., Fountain A.W. (2016). Optimization of Surface-Enhanced Raman Spectroscopy Conditions for Implementation into a Micro fluidic Device for Drug Detection. Anal. Chem..

[19] Pallaoro A., Hoonejani M.R., Braun G.B., Meinhart C.D., Moskovits M. (2015). Rapid Identification by Surface-Enhanced Raman Spectroscopy of Cancer Cells at Low Concentrations Flowing in a Microfluidic Channel. ACS Nano.

[20] Le Ru E.C., Blackie E.J., Meyer M., Etchegoin P.G. (2007). Surface Enhanced Raman Scattering Enhancement Factors: A Comprehensive Study. J. Phys. Chem. C.

[21] Blackie E. (2010). Quantification of the Enhancement Factor in Surface-Enhanced Raman Scattering. Ph.D. Thesis.

[22] Pilot R., Signorini R., Durante C., Orian L. (2019). A Review on Surface-Enhanced Raman Scattering. Biosensors.

[23] Born M., Wolf E. (1970). Basic Properties of the Electromagnetic Field. Principles of Optics: Electromagnetic Theory of Propagation, Interference and Diffraction of Light.

[24] Henyey L.G., Greenstein J.L. (1941). Diffuse radiation in the galaxy. Astrophys. J..

[25] Michels R., Foschum F., Kienle A. (2008). Optical properties of fat emulsions. Opt. Express.

[26] Binzoni T., Leung T.S., Gandjbakhche A.H., Rüfenacht D., Delpy D.T. (2006). The use of the Henyey-Greenstein phase function in Monte Carlo simulations in biomedical optics. Phys. Med. Biol..

[27] Berk A., Bernstein L.S., Anderson G.P., Acharya P.K., Robertson D.C., Chetwynd J.H., Adler-Golden S.M. (1998). MODTRAN cloud and multiple scattering upgrades with application to AVIRIS. Remote Sens. Environ..

[28] Nafie L., Stein P., Fanconi B., Peticolas W.L. (1970). Angular Dependence of Raman Scattering Intensity. J. Chem. Phys..

[29] Gangadhara S. Zemax OpticStudio Knowledgebase - Bulk Scattering with the Rayleigh Model. https://customers.zemax.com/os/resources/learn/knowledgebase/bulk-scattering-with-the-rayleigh-model.

[30] Barycka I., Hołodnik B., Misiuk A. (1981). NiP as a New Material for Thick Film Technology. Electrocompon. Sci. Technol..

[31] Schmidt M.S., Hübner J., Boisen A. (2012). Large area fabrication of leaning silicon nanopillars for Surface Enhanced Raman Spectroscopy. Adv. Mater..

[32] Li M., Zhao F., Zeng J., Qi J., Lu J., Shih W. (2014). Microfluidic surface-enhanced Raman scattering sensor with monolithically integrated nanoporous gold disk arrays for rapid and label-free biomolecular detection. J. Biomed. Opt..

[33] Tran C.T.K., Tran H.T.T., Bui H.T.T., Dang T.Q., Nguyen L.Q. (2017). Determination of low level nitrate/nitrite contamination using SERS-active Ag/ITO substrates coupled to a self-designed Raman spectroscopy system. J. Sci. Adv. Mater. Devices.

[34] Frost R.L., Kristof J., Rintoul L., Kloprogge J.T. (2000). Raman spectroscopy of urea and urea-intercalated kaolinites at 77 K. Spectrochim. Acta Part A.

[35] Frost R.L., Erickson K.L. (2005). Vibrational spectroscopic study of the nitrate containing hydrotalcite Mbobomkulite. Spectrochim. Acta Part A.

[36] Zhang J., Li X., Sun X., Li Y. (2005). Surface Enhanced Raman Scattering Effects of Silver Colloids with Different Shapes. J. Phys. Chem. B.

[37] Luo Z., Fang Y., Yao J. (2007). A New Approach for Non-destructive Detection of Dye Molecules by Combination of Terahertz Time-domain Spectra and Raman. Spectra. Trends Appl. Sci. Res..

