# Supplementary material for: A Tunable Freeform-Segmented Reflector in a Microfluidic System for Conventional and Surface-Enhanced Raman Spectroscopy
Source: Sensors (Basel). 2020 Feb 25;20(5):1250. doi: 10.3390/s20051250 (PMC7085544; doi:10.3390/s20051250)
Supplement: Supplementary file 1 [file sensors-20-01250-s001.pdf]

Supporting Information

Numerical Approach to Calculate the Shape of the Freeform-Segmented Reflector

The Middle Concave Segment Design

There are two methods to design the middle segment, either by conventional sequential ray tracing to reduce the various aberrations and obtain an optimal surface, or by a numerical approach to calculate the surface profile directly. We will not discuss the ray-tracing-based optimization method in this paper but focus on the numerical approach.

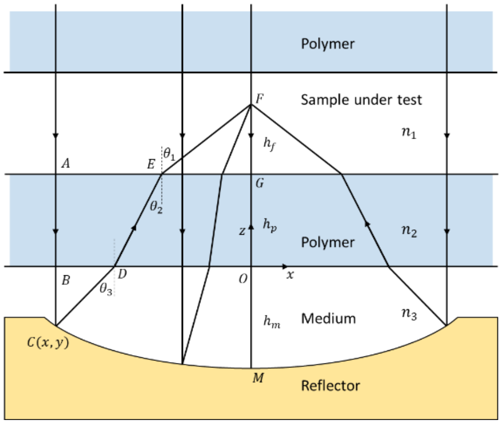

Figure S1. Surface profile of the concave segment, the parallel rays are reflected through the bottom layer of microfluidic chip into the channel layer.

As shown in Figure 1, the refractive index of the sample under test in the channel and the bottom polymer layer are n_1 and n_2, respectively. Additionally, the medium between the bottom polymer layer and the reflector has a refractive index of n_3.

The excitation ray AB that is perpendicular to the microfluidic chip is reflected by the concave surface at point C, enters the bottom layer of the microfluidic chip at point D and is refracted to the upper surface of the polymer layer at point E. The angle of incidence, angle of refraction and angle of emergence of the reflected ray at the bottom polymer layer are θ_1, θ_2, θ_3 respectively. F is the focal point for all the excitation rays. Points O and G are the intersections of the central ray with the bottom and upper surfaces of the bottom polymer layer, respectively. M is the lowest point of the reflector's surface.

Suppose the focal height, or the distance between the upper surface of the polymer layer and the focal point, is h_f. The thickness of polymer layer is h_p, the distance between the lower surface of the polymer layer and the reflector is h_m.

According to the Snell's law

$$n_1 \sin\theta_1 = n_2 \sin\theta_2 = n_3 \sin\theta_3 \tag{1}$$

As stated by Fermat's principle, the optical path length of all rays leaving the polymer layer of plane x and focusing to point F is a constant, that

$$n_3 \cdot (BC + CD) + n_2 \cdot DE + n_1 \cdot EF = 2n_3 \cdot h_m + n_2 \cdot h_p + n_1 \cdot h_f \tag{2}$$

Let's construct a Cartesian coordinates system with point O as the origin and the central ray OF as the z-axis. Then the point C on the surface of the reflector can be represented by the coordinates (x, z). And we can also get the following geometric relationships

$$-x = BC \cdot \tan\theta_3 + h_p \cdot \tan\theta_2 + h_f \cdot \tan\theta_1 \tag{3}$$

$$-z = BC \tag{4}$$

$$CD = -z/\cos\theta_3 \tag{5}$$

$$DE = h_p/\cos\theta_2 \tag{6}$$

$$EF = h_f/\cos\theta_1 \tag{7}$$

When inserting the equations (3)-(7) into the equation (2) we get

$$z = \left[n_2 h_p\left(\frac{1}{cos\,\theta_2} - 1\right) + n_1 h_f\left(\frac{1}{cos\,\theta_1} - 1\right) - 2n_3 \cdot h_m\right] / \left[n_3 \cdot \left(1 + \frac{1}{cos\,\theta_3}\right)\right] \tag{8}$$

Also, the abscissa of the surface x is given by

$$x = z \cdot \tan\theta_3 - h_p \cdot \tan\theta_2 - h_f \cdot \tan\theta_1 \tag{9}$$

Because θ_2 and θ_3 can be represented by θ_1 according to equation (1), and as demonstrated by equations (8) and (9), we notice that once h_f, h_p and h_m are confirmed, the surface profile of the concave segment is a function of the angle θ_1. If we fill the same medium as n_1 in the space between the polymer layer and the reflector ($n_1 = n_3$), θ_1 becomes equal to θ_3. The focal height h_f will change proportionally with respect to the height of reflector h_m, making the system tunable in the vertical direction with a stationary spot size. This is crucial as we can place the focal point in the very middle of the channel to minimize the Raman background from the polymer or keep it exactly on the nano-structures of a SERS substrate maximizing the SERS enhancement. Since most of the sample solutions are aqueous, and many other organic solvents such as ethanol or acetone have a similar refractive index as water, therefore we use water as the medium between the reflector and polymer layer ($n_1 = n_3 = n_{water}$).

The Center and Marginal Segment Design

The center and marginal segments are also directly designed by a numerical approach. Figure 2 illustrates the surface profile of all three segments. In this step we use the same Cartesian coordinate system as mentioned before.

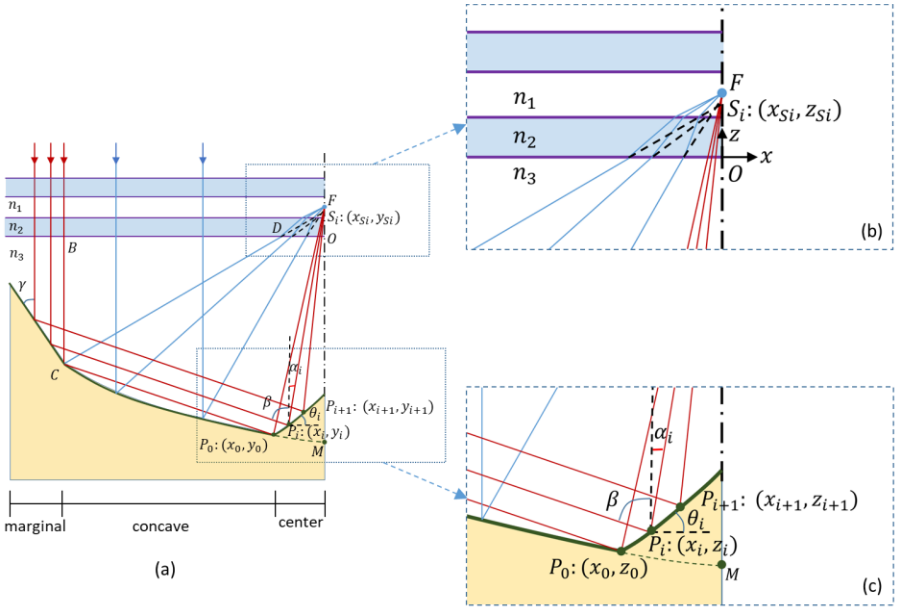

Figure S2. Schematic design of all segments: center, concave and marginal. (a) general view. (b) zoom-in view of the rays in the microfluidic channel. (c) zoom-in view of the rays interacting with the center segment.

Assume $S_i(x_{si}, z_{si})$ is the virtual focal point of a reflected ray by the concave surface, which refers to the intersection of the extension of a reflected ray and the optical axis. Note that S_i is different from the actual focal point F, which can be obtained by ray tracing of the reflected rays according to the surface shape of the concave segment. $P_i(x_i, z_i)$ is a random point of the center segment. S_iP_i is the relevant ray that passes through the same focal point of the concave segment. The angle of inclination of the center segment at point P_i is θ_i. The angle between ray S_iP_i and the optical axis is α_i, and the angle between ray S_iP_i and the reflected ray by center segment is β. β is constant for all rays. The angle of inclination of the marginal segment is γ. M is the end point of the concave segment profile.

For the ray S_iP_i, its slope is given by

$$\tan \alpha_i = (x_i - x_{Si})/(z_i - z_{Si}) \quad (10)$$

For the surface of center segment at point P_i, the angle of inclination θ_i is given by

$$\theta_i = (\beta - \alpha_i)/2 \quad (11)$$

If we describe the center segment by

$$z = f(x) \quad (12)$$

Then the slope of center segment is given by

$$\tan \theta_i = (z_{i+1} - z_i)/(x_{i+1} - x_i) \quad (13)$$

The numerical calculation of the surface profile of the center segment starts from the initial point P_0. The radius of the center segment $|x_0|$ is determined by the lateral dimension of the SERS substrate to make sure the entire surface area of the SERS substrate can be covered by the center segment. The angle value of β can firstly be derived from the inclination of ray P_0M. Then $\tan \alpha_0$, the slops of the ray S_0P_0, is obtained by equation (10). The derived angle value of α_0 is introduced to equation (11) to get the value of θ_0. In a next step, the coordinate of $P_1(x_1, z_1)$ is calculated as the intersection point of ray S_1P_1 and the curve P_0P_1. The overall profile of the center segment is calculated by iteration of this process from the coordinates of P_1 to P_i.

The profile of the marginal segment is a straight line with an angle of inclination that is given by

$$\gamma = (\pi - \beta)/2 \tag{14}$$

Normally, the thickness of the polymer layer for the microfluidic chip should be larger than 200μm to guarantee mechanical strength. To reduce fabrication complexity, we introduce a 1mm thick commercial polymer plate in our design, which is identical to many other commercial microfluidic chips. We accomplished the segmented freeform design for two configurations with a diameter of the center segment of 5mm and 10mm respectively, as shown in Figure 3. The numerical aperture of both configurations is 1.15, making their collecting efficiencies close to that of a 100× magnification objective lens. The overall diameter of the segmented reflector increases from 30 mm to 38 mm when the diameter of the center segment increases from 5 mm to 10 mm. The sag of the whole reflector also increases with a larger diameter of the center segment. As a result, the tunability of the system decreases from 4mm to 0.5mm. Since the 5mm diameter of the center segment is wide enough to cover the SERS substrate for our application, we utilize the 5mm center segment design in the following non-sequential ray tracing simulation, fabrication and experiments.

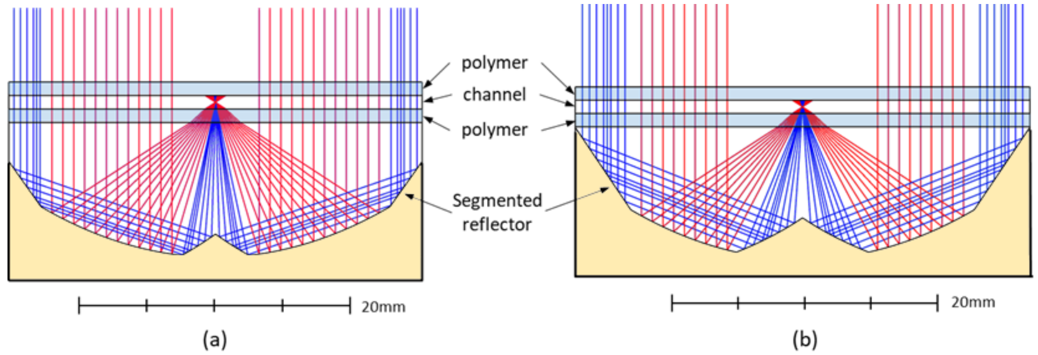

Figure S3. Two configurations of reflector and microfluidic chip design, the diameters of center segment for the (a) and (b) configuration are 5 mm and 10 mm, respectively, and the overall diameters are 30 mm and 38 mm. NA of both configurations is 1.15. Moreover, the NA of the center segment is 0.34 and 0.45 respectively. The blue and red rays refer to the incident light that interact with different segments.

The Henyey-Greenstein Model for the Non-Sequential Simulation

In the Henyey-Greenstein model [24], the scattered light has the following angular distribution $\rho(\theta)$

$$\rho(\theta) = \frac{1}{4\pi}\frac{1-g^2}{(1+g^2-2g\cos\theta)^{\frac{3}{2}}} \tag{1}$$

Where, θ is the angle of scattered light with respect to the incident light. g is an asymmetric factor ranging from -1 to 1. According to this equation, the forward scattering is dominant when g>0. While g<0, backscattering predominates. For an oriented molecule or crystal, the angular distribution of Raman scattering is heterogeneous [28]. But in most cases, as the molecules in the sample are randomly oriented, the Raman scattering is nearly isotropic in 4π steradian. Therefore, we set g=0 to simplify the simulation.

The other two important parameters of the Henyey-Greenstein model in Zemax OpticStudio are transmission and mean free path [29]. The transmission parameter indicates how much of the incident light is attenuated during scattering. The mean free path (M) refers to the probability of a ray being scattered. In Zemax OpticStudio, the integrated probability of a ray traveling a distance x within the medium and undergoing scattering is given by [29]

$$p(x) = 1.0 - e^{-\frac{x}{M}} \tag{2}$$

The larger the value of M, the lower the probability that the incident light will be scattered.

Spectra Measured with our microfluidic system

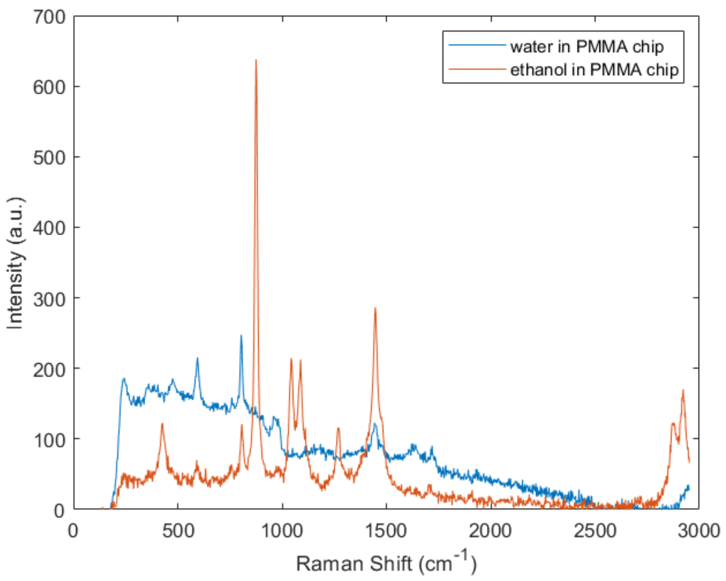

Figure S4. Raman response of water and ethanol filled in the fluidic channel. The spectrum of water is generally used to correct the baseline for the aqueous solutions under test.

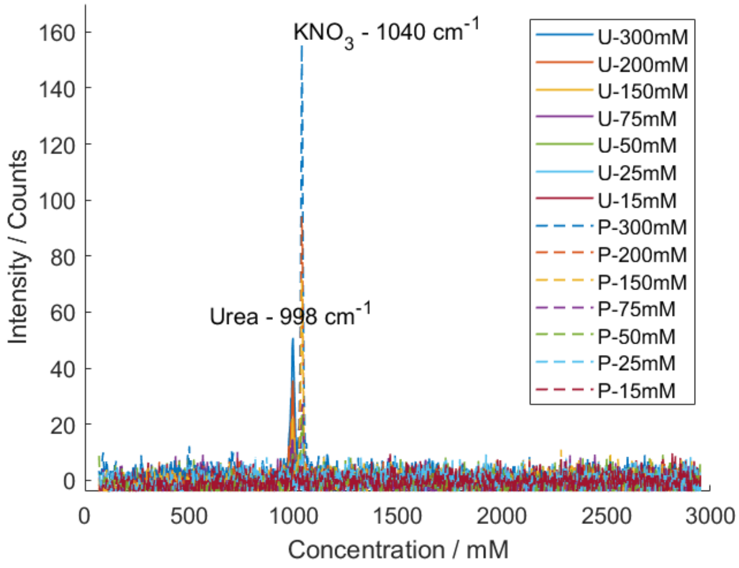

Figure S5. Raman spectra of urea (U) and potassium nitrate solutions (P) with different concentrations. Baseline is corrected by subtracting the reference spectra of water. Each spectrum is obtained over 10 measurements.

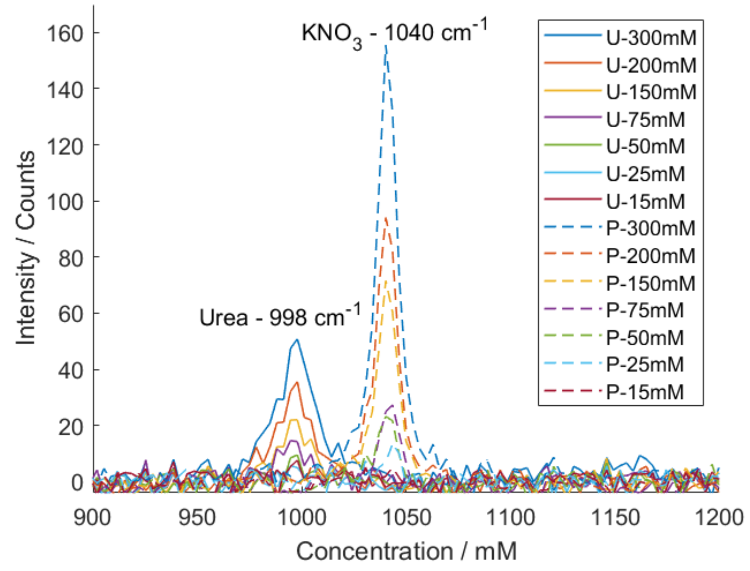

Figure S6. Main Raman bands of urea (U) and potassium nitrate solutions (P) with different concentrations. The baseline is corrected by subtracting the reference spectrum of water. Each spectrum is obtained over 10 measurements.

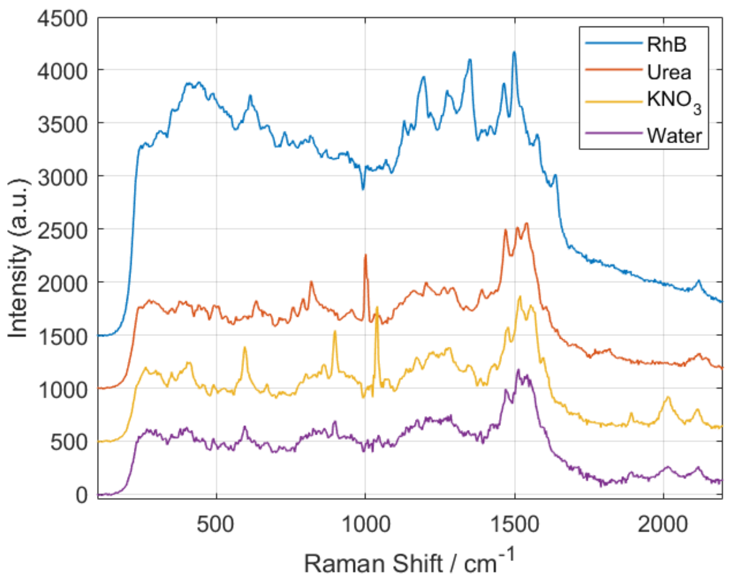

Figure S7. Raman spectra of 0.01mM Rhodamine B (RhB), 0.1mM urea, 0.1mM potassium nitrate solutions and water obtained with our microfluidic system in combination with the SERS substrate. Each spectrum is obtained over 10 measurements.

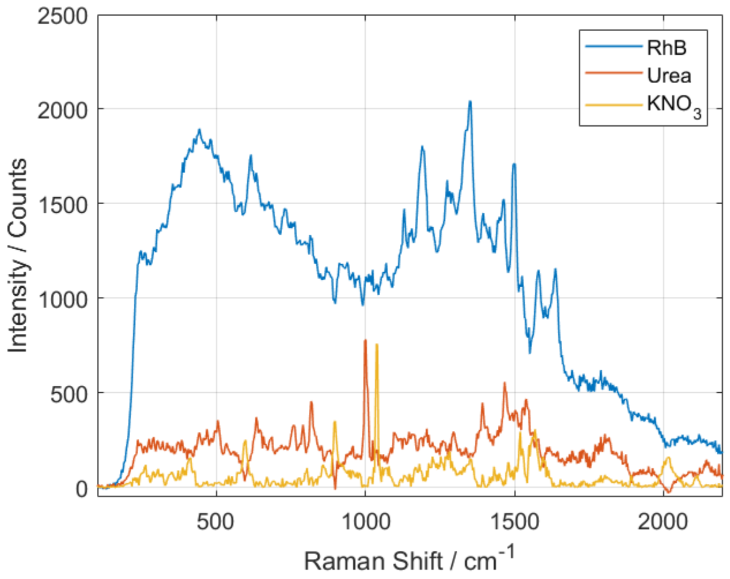

Figure S8. Raman spectra of 0.01mM Rhodamine B (RhB), 0.1mM urea, 0.1mM potassium nitrate solutions obtained with our microfluidic system in combination with the SERS substrate. The baseline is corrected by subtracting the reference spectrum of water. Each spectrum is obtained over 10 measurements.
